# Endovascular covered stenting for the management of post-percutaneous nephrolithotomy renal pseudoaneurysm: a case report

**DOI:** 10.1186/1752-1947-4-316

**Published:** 2010-09-23

**Authors:** Prodromos Philippou, Konstantinos Moraitis, Tamer El-Husseiny, Hassan Wazait, Junaid Masood, Noor Buchholz

**Affiliations:** 1 Department of Urology, Barts and The London NHS Trust, Smithfield, London EC1A 7BE, UK

## Abstract

**Introduction:**

Intrarenal pseudoaneurysm is a rare, yet clinically significant, complication of percutaneous nephrolithotomy. A high index of clinical suspicion is necessary in order to recognize pseudoaneurysm as the cause of delayed bleeding after percutaneous nephrolithotomy and angiography confirms the diagnosis which allows endovascular management.

**Case presentation:**

We present a case of a 65-year old Caucasian woman who underwent percutaneous nephrolithotomy in the supine position for a two centimetre renal calculus. The postoperative course was complicated by persistent bleeding due to a renal pseudoaneurysm. The vascular lesion was successfully managed by endovascular exclusion through the use of a covered stent graft. We report the first successful use of this method for the management of iatrogenic pseudoaneurysm in a branch of the left renal artery and we focus on the imaging findings, technical details, advantages and limitations of this technique.

**Conclusion:**

As a result of its high efficacy, interventional radiology has largely replaced open surgery for the management of renal pseudoaneurysm related to percutaneous nephrolithotomy. Recent technical advancements have allowed the use of covered stent grafts as an alternative to embolisation for the angiographic management of visceral artery pseudoaneurysm located in other organs. This novel technique allows the endovascular exclusion of the pseudoaneurysm, without compromising arterial supply to the end-structures - an advantage of critical importance in organs supplied by segmental arteries - in the absence of collateral vasculature, such as the kidney.

## Introduction

Renal pseudoaneurysm (PA) is a rare, yet clinically significant, cause of delayed bleeding following percutaneous nephrolithotomy (PCNL). According to the current literature, the reported incidence of intrarenal PA following PCNL is low (0.6%-1%) [1,2]. A high index of clinical suspicion is of the utmost importance, while angiography is usually necessary in order to identify the source of bleeding and treat the vascular injury. Angiographic management - usually by superselective embolisation of the injured vessel - has success rates that exceed 90% and has largely replaced the need for open surgery [2,3]. We report a unique case of a PCNL-related renal PA which was successfully managed by a covered stent graft exclusion, a technique that was recently developed for the management of PAs located elsewhere.

## Case presentation

**Figure 1:**
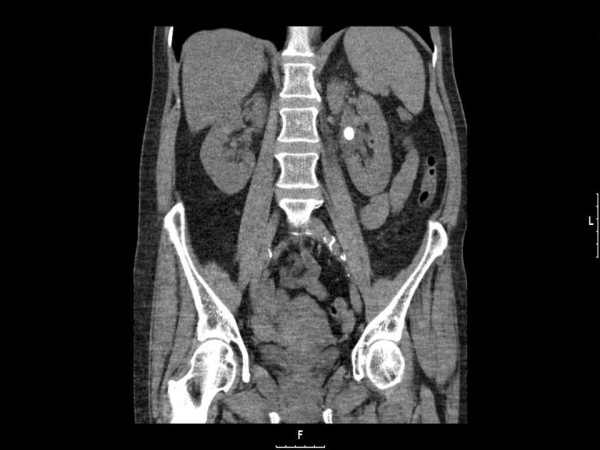
**Computed tomography of the kidneys, ureter and bladder, prior to percutaneous nephrolithotomy**. Note a two centimetre stone located at the left renal pelvis.

A 65-year old Caucasian woman, with a two centimetre calculus located in the pelvis of the left kidney (Figure 1), underwent supine PCNL. Percutaneous access was achieved through the middle calyx: the procedure was uneventful and the intra-operative blood loss was minimal. At the end of the procedure, stone-free status was achieved and a Malekot-type 22 Fr nephrostomy tube was left *in situ*.

**Figure 2:**
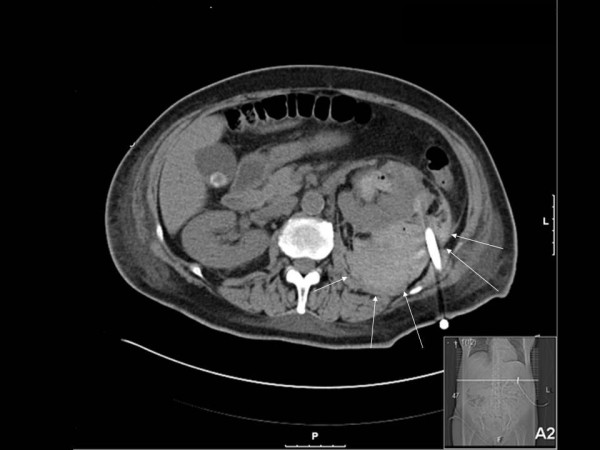
**Abdominal computed tomography scan prior to angiography**. A large left perinephric haematoma with active extravasation of contrast is identified (arrows).

On the fourth post-operative day, the patient developed gross hematuria and severe pain of the left loin. A significant drop in the hemoglobin level was noted (from 9.5 g/dL to 6.8 g/dL) but she remained hemodynamically stable and the coagulation parameters were within normal limits. She was initially treated conservatively with bed rest and transfusions but gross hematuria persisted. An abdominal computed tomography (CT) scan (Figure 2) revealed the presence of a large left perinephric hematoma with active extravasation of contrast. An urgent selective left renal angiogram was arranged in order to achieve endovascular control of the bleeding vessel.

**Figure 3:**
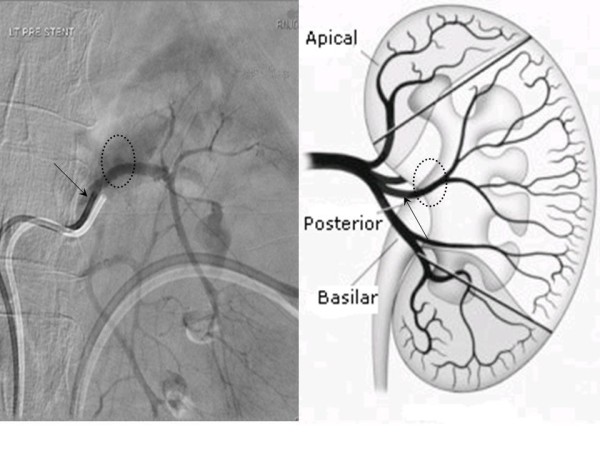
**Selective angiogram of the left renal artery**. A pseudoaneurysm is noted, arising from a branch of the posterior division of the left renal artery, with active extravasation of contrast (dotted circle). Selective catheterisation and embolisation of the bleeding branch was technically not feasible as advancement of the endovascular catheter was not possible beyond the main stem of the posterior branch of the renal artery (arrow).

**Figure 4:**
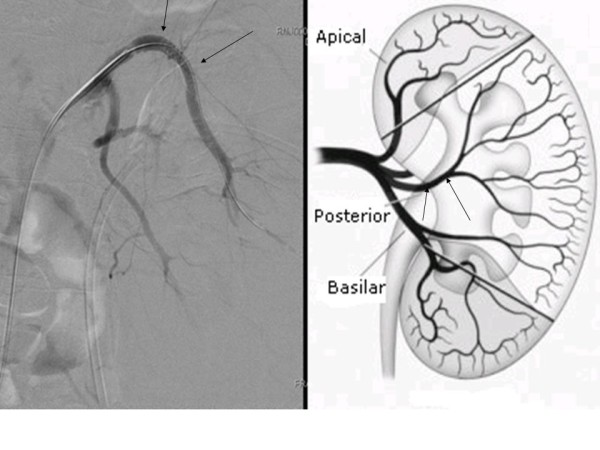
**Control angiogram at the end of the procedure**. A 6 × 19 mm self-expandable fluency covered stent was advanced over a guidewire and deployed in order to achieve endovascular exclusion of the pseudoaneurysm (PA; arrows). The control angiogram at the end of the procedure revealed the absence of opacification of the PA, with appropriate preservation of renal parenchymal perfusion.

Access was achieved through the right common femoral artery and selective catheterisation of the left renal artery was performed. On angiography, a PA was noted, arising from a branch of the posterior division of the left renal artery, with active extravasation of contrast (Figure 3). Selective catheterisation and embolisation of the bleeding branch was technically not feasible. Embolisation of a more proximal arterial branch was considered inappropriate due to the associated risk of more extensive renal parenchymal ischemia. In order to overcome these limitations, a 6 mm × 19 mm self-expandable Fluency covered stent™ (Bard, New Jersey, USA) was advanced over a guidewire and deployed to achieve endovascular exclusion of the PA. A control angiogram at the end of the procedure revealed the absence of opacification of the PA, with the appropriate preservation of renal parenchymal perfusion (Figure 4).

Twenty-four hours later, hematuria ceased and the patient remained hemodynamically stable. An abdominal CT angiogram was performed in order to enable us to evaluate the result of the endovascular manipulation. Uniform global enhancement of the renal parenchyma was noted. There were no signs of active contrast extravasation or opacification of an aneurysmal cavity.

**Figure 5:**
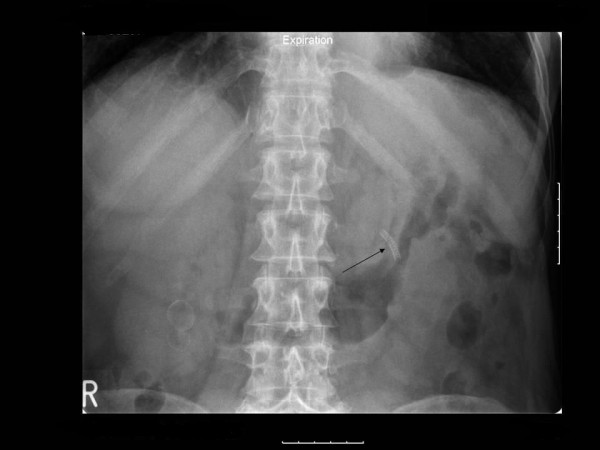
**A plain abdominal X-ray film**. Six weeks postoperatively, a plain abdominal X-ray confirmed stone-free status and the presence of a covered stent graft in the anatomic location corresponding to the left kidney (arrow).

The woman was discharged 48 hours later and a plain abdominal X-ray film, which was done six weeks later, confirmed stone-free status and the presence of a covered stent graft in the anatomic location corresponding to the left kidney (Figure 5).

## Discussion

Percutaneous access to the upper urinary tract was first described in 1955, while PCNL was introduced 20 years later [4]. Since then, PCNL has undergone many refinements and is considered to be the current method of choice for the management of large, or otherwise complex, renal stone disease [4,5]. Despite being a minimally-invasive technique, PCNL is associated with clinically-significant bleeding, with transfusion rates in contemporary literature between 5%-18% [6].

Major vascular complications caused by vessel injury during PCNL - namely, PAs or arteriovenous fistula - usually present as delayed postoperative bleeding after a mean delay of eight days [1]. The percutaneous tract disrupts the normal vessel wall and a PA is formed from the tissues surrounding the high-pressure arterial system, resulting in recanalisation between the intravascular and extravascular space that produces a pulsating, encapsulated hematoma. The PA may eventually grow and become unstable, with erosion into the pelvicaliceal system or the perinephric tissue [7]. Srivastava *et al. *identified stone burden as a significant predictor of severe vascular injuries after PCNL [1] and this has now been reproduced by others [2]. Surgical experience was also identified as a significant predictor for clinically significant PCNL-related vascular injuries [2]. Post-PCNL PA is usually located in the peripheral arteries. In our case the tract was done by using the standard technique. The segmental artery was slightly atypically located and the Amplatz sheath and, later, the large bore nephrostomy catheter left *in situ *might have temporarily tamponaded the bleeding. This might explain the absence of significant bleeding intraoperatively and immediately postoperatively.

The diagnosis of intrarenal PA is challenging. Angiography has emerged as the standard but multiple non-invasive tests, such as renal ultrasound, intravenous pyelography, contrast-enhanced CT scanning (with three dimensional reconstruction), magnetic resonance imaging and renal scintigraphy, have been used with moderate success in diagnosing renal artery pseudoaneurysm [8]. The advantages of angiography in this setting include high sensitivity in identifying the PA (which usually appears as a round or oval structure arising from the main renal artery or one of its branches) and the potential to achieve simultaneous endovascular management of these lesions, with success rates exceeding 90% [3]. Superselective embolisation is highly efficient in achieving PA occlusion through the injection of a permanent agent at the fistulous point. Materials such as ethanol, gel foam particles and N-butyl-2-cyanoacrylate [3,7,8] have been successfully used for embolisation. However, embolisation for the management of PA does have some shortcomings, such as possible reflux of embolic material into the normal proximal vessel if the distal branch has not been selectively cannulated and the risk of more generalised ischemia resulting from thrombosis of a main feeding branch [9].

In order to overcome these limitations, covered stent-grafts have been used for the treatment of PA located in branches of visceral arteries, such as the hepatic and splenic artery [9,10]. To date, a total of 17 cases of visceral artery PAs managed by endovascular covered stenting have been described in the medical literature [10]. However, our case represents the first report on the successful use of this method for the management of an iatrogenic PA in a branch of the renal artery This technique allows for the endovascular exclusion of a PA without compromising blood flow to the end-structures, an advantage of critical importance in organs supplied by segmental end-arteries in the absence of collateral vasculature, such as the kidney. The Fluency™ device (Bard, New Jersey, USA) is a carbon coated, expanded polytetrafluoroethylene (PTFE) encapsulated nitinol stent which has two mm of bare metal exposed at each end [11].

An important limiting factor in the use of covered stents is the size and rigidity of the available systems. Covered stents are reserved for lesions located at major arterial branches. They are usually used for arteries that are more than six millimeters in diameter because of the risk of thrombosis when used for smaller vessels [12]. These factors may preclude the use of this technique for the management of lesions involving small-calibre and tortuous renal vessels [12]. Currently, there is a lack of long-term data that support the indiscriminant use of this technique. Embolisation remains the gold standard for the management of post-PCNL PA, especially for lesions located at the distal branches of the renal artery. However, the short-term data regarding the use of the technique for the management of visceral PA located elsewhere are promising. Another issue of concern is the possibility of stenosis at the ends of the stent or within the stent. The use of stents covered with autogenous material or drug-eluding stents may resolve this problem. One of the aims of future research in this field should be the improvement of the profile and longitudinal flexibility of these stents which could facilitate their positioning and deployment, even in complex vascular lesions.

## Conclusion

Expanding worldwide experience has allowed PCNL to become a significant technique with high stone clearance rates and low morbidity. PCNL-related vascular injuries are rare but life-threatening complications. The advances of the endovascular technique have allowed the successful treatment of the vast majority of renal PAs by embolisation, while covered stenting may emerge as a highly effective and safe alternative, allowing the repair of a PA, without compromising arterial supply to the end-structures.

## Abbreviations

CT: computed tomography; PA: pseudoaneurysm; PCNL: percutaneous nephrolithotomy.

## Competing interests

The authors declare that they have no competing interests.

## Consent

Written informed consent was obtained from the patient for publication of this case report and accompanying images. A copy of the written consent is available for review by the Editor-in-Chief of this journal.

## Authors' contributions

PP analyzed and interpreted the patient data, reviewed the literature and was responsible for drafting the manuscript. KM made substantial contributions to the conception, design and acquisition of data. TEH made substantial contributions to the analysis and interpretation of data and revised the study critically for important intellectual content. HW reviewed the current literature and was responsible for the interpretation of the imaging finding. JM made substantial contributions to the conception and design of this study and revised it critically for important intellectual content. NB reviewed the literature, made substantial contributions to the conception and design of this study and revised it critically for important intellectual content. All authors read and approved the final manuscript.
